# Factors Predicting Cybersex Use and Difficulties in Forming Intimate Relationships among Male and Female Users of Cybersex

**DOI:** 10.3389/fpsyt.2015.00054

**Published:** 2015-04-20

**Authors:** Aviv M. Weinstein, Rinat Zolek, Anna Babkin, Koby Cohen, Michel Lejoyeux

**Affiliations:** ^1^Department of Behavioral Sciences, University of Ariel, Ariel, Israel; ^2^Department of Psychiatry, Paris 7 University, Paris, France; ^3^Hospital Bichat Claude Bernard, AP-HP and Maison Blanche Hospital, Paris, France

**Keywords:** sex addiction, pornography, cybersex, intimacy, craving

## Abstract

Sexual addiction otherwise known as compulsive sexual behavior is associated with serious psychosocial problems and risk-taking behavior. This study used the Cybersex addiction test, Craving for pornography questionnaire, and a Questionnaire on intimacy among 267 participants (192 males and 75 females) mean age for males 28.16 (SD = 6.8) and for females 25.5 (SD = 5.13) who were recruited from special sites that are dedicated to pornography and cybersex on the Internet. Results of regression analysis indicated that pornography, gender, and cybersex significantly predicted difficulties in intimacy and it accounted for 66.1% of the variance of rating on the intimacy questionnaire. Second, regression analysis also indicated that craving for pornography, gender, and difficulties in forming intimate relationships significantly predicted frequency of cybersex use and it accounted for 83.7% of the variance in ratings of cybersex use. Third, men had higher scores of frequency of using cybersex than women [*t*(2,224) = 1.97, *p* < 0.05] and higher scores of craving for pornography than women [*t*(2,265) = 3.26, *p* < 0.01] and no higher scores on the questionnaire measuring difficulties in forming intimate relationship than women [*t*(2,224) = 1, *p* = 0.32]. These findings support previous evidence for sex differences in compulsive sexual behavior.

## Introduction

Sex addiction otherwise known as Compulsive sexual behavior, has been associated with serious psychosocial problems and risk-taking behaviors. This behavior has not been recognized as a disorder that merits inclusion in the DSM (1) see Ref. (2–4) for recent reviews. Despite different views about pathological characteristics of sexual addiction there is an agreement that this is a progressive relapsing condition, which does not merely refer to sexual lifestyle that is socially deviant (2–4). Recently, the American Psychiatric Association Board of Trustees rejected several proposals for the new disorder and therefore sexual addiction does not appear in the DSM-5. Even though clinicians have been treating the disorder, the Board of Trustees estimated that there was not enough research to consider adding the disorder to Section 3 (disorders that require further research) of the DSM-5 (5).

Sex addiction is associated with behaviors such as constantly seeking new sexual partners, having frequent sexual encounters, engaging in compulsive masturbation, and frequently using pornography. Despite of efforts to reduce or stop excessive sexual behaviors individuals with sex addiction find it difficult to stop and they engage in risky sexual activities, pay for sexual services, and resist behavioral changes to avert risk of HIV (6–9). Cognitive and emotional symptoms include obsessive thoughts of sex, feelings of guilt about excessive sexual behavior, the desire to escape from or suppress unpleasant emotions, loneliness, boredom, low self-esteem, shame, secrecy regarding sexual behaviors, rationalization about the continuation of sexual behaviors, indifference toward a regular sexual partner, a preference for anonymous sex, a tendency to disconnect intimacy from sex, and an absence of control in many aspects of life (7, 8, 10, 11). Finally, some studies find that sexual addiction is associated with or in response to dysphoric affect (9, 12–16) or stressful life events (17).

Pornography has a decisive role in establishing basic assumptions about identity, sexuality, women’s worth, nature of relationships, and their long-term addictive effects. The easy availability to pornographic content on the Internet go beyond human imagination and fantasy and enables graphic interactive encounters that fulfill urges for nudity and sexual encounters with available women always for pleasure with minimal implications and temporary encounters. Online sexual activity includes viewing and downloading pornography, visiting sex shops for sexual aids and toys, advertising or hiring sex workers on the Internet, seeking sex education information, locating sex contacts, and interacting with sexual subcultures or communities (18). Exposure to pornography results in reduced self-esteem and body image satisfaction, increased sense of vulnerability to violence, and an increased sense of defenselessness in women, and in men in reward for displays of hyper masculinity and trivializing or excusing violence against women (19). These effects are seen not only in men’s perceptions of women but also in women’s own perceptions of themselves. Pornographic norms for gender relationships and sexuality infuse many forms of media, such as music videos, reality television shows, even children’s toys. Thus, it becomes difficult to distinguish pornography’s specific effects from those of the general climate of gender inequality in the culture of pornography (20).

Cybersex usually involves watching, downloading, and online trading of pornography or connecting to chat rooms using role plays and fantasy for men (21) and this space enables people to explore and investigate their sexual urges and private fantasies online (22). Cybersex addicts tend to suffer from poor impulse control and often have a history of multiple addictions to alcohol, tobacco, drugs, gambling, food, or sex. If an online user already suffers from a history of sexual addiction, cybersex serves as another outlet for gratification that feeds a previous problem. However, new research has found that over 65% of cybersex addicts have no history of sexual addiction (23). There are studies showing that cybersex negatively affects the patient, the spouse, and the family (24, 25). Other studies have found that males use cybersex for mood management (26, 27). Although cybersex can be used as an outlet for sexual activity there is therefore no evidence that those who use it are sexually addicted. It is important to investigate the relationship between pornography and cybersex and to ascertain their effects on the ability to form intimate relationships in men and women.

Recent studies by Laier and Brand (28, 29) explain the use of pornography and cybersex as means of sexual arousal and gratification. Furthermore, Laier and Brand (30), described a model on the development and maintenance of cybersex addiction which is based on the model for Internet addiction introduced by Brand et al. (31). These models support the arguments for the link between pornography and cybersex.

Consistent with previous studies and models on sex addiction (28–31), we have investigated the frequency of cybersex use, craving for pornography and the ability to form intimate relationships among men and women who use pornography and cybersex on the Internet. In accordance with findings of previous research, we have predicted that frequency of using cybersex, craving for pornography would predict difficulty in intimacy in men and women who use cybersex. Second, we have predicted that sex, craving for pornography and difficulties in intimacy would predict frequency of cybersex use. Third, we have predicted that there would be sex differences in the frequency of use of cybersex and craving for pornography.

## Procedure

### Participants

The participants of this study were recruited from forums on the Internet that are dedicated to pornography and cybersex in order to satisfy sexual curiosity and arousal.

Men and women were approached on the websites and were asked to fill in questionnaires and send them by mail to the investigators. Questionnaires were anonymous and there were no means for assessing deception by the participants. Inclusion criteria for compulsive sexual behavior were males and females who use the Internet for sex purpose. From the original sample of 272, five participants did not meet inclusion criteria and were removed from the sample and 267 participants remained. The sample included 192 men (72%) and 75 women (28%) with mean age for males 28 years and 2 months (SD = 6.8) and for females 25 years and 6 months (SD = 5.13). Men were significantly older than women in this sample [*t*(2,265) = 3.61; *p* < 0.01]. Education attainments were 6.7% with university Master’s degree, 40.4% with university Bachelor degree, 27.7% high school education, 23.6% further education after high school, 1.5% with elementary school education. Employment status of the participants included 40.4% full-time employment, 35.6% part-time employment, and 24% unemployed. Marital status was 14.2% married, 57.7% bachelors, 23.6% in relationship but not married, 4% separated, 4.1% divorced. Most of the participants lived in the city (83.5%) and 16.5% lived in rural areas. Most of the participants were Jewish (91%), 2.2% Muslims, 4% Christians, and 2.8% others.

### Questionnaires

(1) *Demographic questionnaire* including items on age, sex, education, employment status, marital status, type of living (urban or rural), and religion.
(2) *Cybersex addiction test* (23), which consists of 20 questions about cybersex addiction including pornography. For example, rate the frequency that you neglect your duties in order to spend more time in cybersex, the frequency that you prefer cybersex on intimacy with your partner, the frequency that you spend time in chat rooms and private conversations in order to find partners for cybersex, the frequency that people complain about the time that you spend online, etc.
  The scale is from 0 to 5 where 0 is “not applicable” and 5 is “always.” The Cronbach measure of internal validity of the questionnaire was α = 0.95. Participants were divided into four groups non-addicted (score 0–30), moderately addicted (31–49), medium addiction (50–79), and severely addicted (80–100).
(3) *Craving for pornography questionnaire* (32), which consists of 20 questions about perceived control in using pornography, changes in mood, psychophysiological activity, and intention for using pornography. The scale is from 1 (“do not agree at all”) to 7 (“agree very much”). The questionnaire was validated by Kraus (32) on US students and it has a Cronbach internal reliability of α = 0.94. Scores vary from low levels of craving for pornography (0–20) and high craving for pornography (100–140).
(4) *Questionnaire on difficulties in intimacy* (33), which consists of 12 questions including 4 questions on fear of abandonment, 4 on fear of exposure, and 4 on shame and fear of rejection. The questionnaire has been widely used for research on psychosocial intimacy and for couple treatment. The scale is from 0 (“does not describe me”) to 4 (“definitely describes me”). The questionnaire has a Cronbach internal reliability of α = 0.85. Scores vary between 0 = no problems in intimacy and 44 = lots of problems in intimacy.

### Procedure

The questionnaires were filled in online using a form that was created through Google Drive and was sent as a link on email messages to members in groups and forums on pornography and cybersex. Those who responded filled in the questionnaires and informed consent forms while privacy and anonymity were maintained. The study was approved by the Institutional Review Board (IRB-Helsinki committee) of the University of Ariel in Israel.

### Statistical analysis

(1) Descriptive statistics of male and female participants on the questionnaires measuring frequency of cybersex, craving for pornography and difficulties in intimacy was performed.
(2) Regression analysis:
  A stepwise regression analysis was performed with measures of intimacy as a dependent variable. In the first step, craving for pornography was entered; in the second step, gender was entered; and in the third step, frequency of cybersex use entered as independent variables.
(3) Comparison of questionnaire measures according to gender and level of use of cybersex:
  (1) Male and female participants were compared on measures of the questionnaires measuring frequency of cybersex, craving for pornography, and difficulties in intimacy.
  (2) All participants were divided into three groups according to their level of frequency of cybersex use “high,” “medium,” and “low.” An analysis of variance (ANOVA) of the factors of frequency of cybersex, craving for pornography, ratings of intimacy, and gender was performed. *Post hoc* comparisons of questionnaire measures in all groups were performed with Bonferroni corrections for multiple comparisons.
(4) A Pearson correlational analysis between frequency of using cybersex, craving for pornography, and difficulties in forming intimate relationship scores was performed in all participants also separate in men and women.

## Results

### Descriptive statistics

Overall, mean scores on the frequency of cybersex questionnaire (*n* = 226) were 22.65 (SD = 19.38) (score range 0–100), craving for pornography (*n* = 267) 52.47 (SD = 26.9) (score range 20–140), and questionnaire on difficulties in intimacy (*n* = 267) were 14.59 (SD = 9.22) (score range 0–44).

### Regression analysis of all variables

The results of the regression analysis using intimacy ratings as a dependent variable, indicated that the three variables of pornography, gender, and cybersex were significant and they all accounted for 66.1% of the variance of ratings on the intimacy questionnaire. Craving for pornography accounted for 29.3% of the variance, frequency of cybersex accounted for 20% of the variance, and gender accounted for 16.8% of the variance.

The results of the regression analysis using cybersex frequency as a dependent variable, indicated that the three variables of pornography, gender, and cybersex were significant and they all accounted for 83% of the variance of the intimacy questionnaire. Craving for pornography accounted for 58.8% of the variance, intimacy accounted for 13.4% of the variance, and gender accounted for 11.5% of the variance.

See Table 1 for results of the regression analyses.

**Table 1:** **(A) Regression analysis of the effects of pornography, gender, and cybersex addiction scores on intimacy in all participants (*n* = 267); (B) regression analysis of the effects of pornography, gender, and intimacy on cybersex addiction scores in all participants (*n* = 267)**.

Variable	*B*	SE	β	*t* Value	*p* Value
**(A)**^a^
Pornography	0.100	0.02	0.29	3.96	0.0001
Gender	3.43	1.16	0.16	2.95	0.01
Cybersex	0.09	0.03	0.20	2.68	0.01
**(B)**^b^
Pornography	0.43	0.04	0.59	11.62	0.0001
Gender	-5.013	2.03	-0.12	-2.46	0.01
Intimacy	0.284	0.11	0.13	2.69	0.01

*^a^*F*(3,263) = 21.5, *p* < 0.001, *R*^2^ = 0.197*.

*^b^*F*(3,263) = 75.65, *p* < 0.0001, *R*^2^ = 0.463*.

### Comparison of questionnaire measures according to gender

(1) A comparison of scores of frequency of using cybersex between men and women found that men had a higher score (Mean = 24.02, SD = 19.25) than women (Mean = 17.98, SD = 19.31); *t*(2,224) = 1.97, *p* < 0.05.
(2) A comparison of craving for pornography scores between men and women found that men had a higher score (Mean = 55.77, SD = 27.35) than women (Mean = 44.03, SD = 23.86); *t*(2,265) = 3.26, *p* < 0.01.
(3) A comparison of the questionnaire on difficulties in forming intimate relationship between men and women found no significant difference between scores of men (Mean = 15.56, SD = 8.86) and women (Mean = 13.85, SD = 9.45); *t*(2,224) = 1, *p* = 0.32.

Table 2 shows means and (SD) of males and females on all questionnaires and comparisons between men and women using *t*-tests on all measures.

**Table 2:** **Means and (SD) of males and females on all questionnaires**.

	Cybersex	Pornography	Intimacy
Men	Mean = 24.02, SD = 19.25	Mean = 55.77, SD = 27.35	Mean = 15.56, SD = 8.86
Women	Mean = 17.98, SD = 19.31	Mean = 44.03, SD = 23.86	Mean = 13.85, SD = 9.45
Comparison	*t*(2,224) = 1.97, *p* < 0.05	*t*(2,265) = 3.26, *p* < 0.01	*t*(2,224) = 1, *p* = 0.32

**Figure 1:**
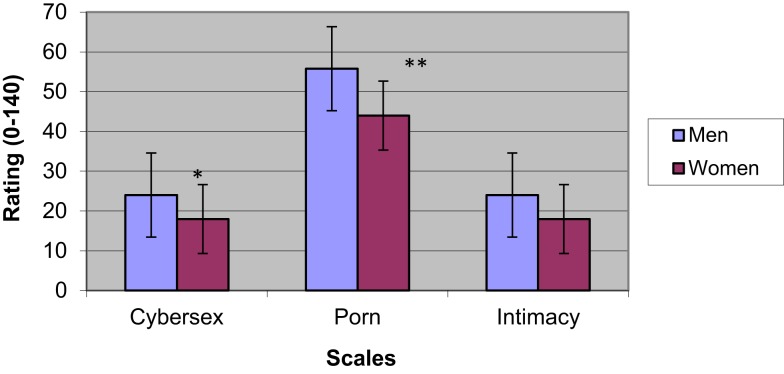
**Questionnaire ratings of cybersex, porn and intimacy – a comparison between men and women**. *p < 0.05; **p < 0.01; ***p < 0.001.

Figure 1 shows differences between men and women on measures of addiction to cybersex, craving for pornography, and difficulty in forming intimate relationships.

### An analysis of questionnaire measures according to level of cybersex use

All participants were divided into three groups according to their level of frequency of cybersex use: participants with 1 standard deviation above mean cybersex score were included in the “high frequency cybersex group” (*n* = 54 score above 36), participants with <1 SD above mean cybersex score and more than 1 SD below mean cybersex score were included in the “medium frequency cybersex group” (*n* = 172 < 1 score < 36) and participants with <1 SD below mean cybersex score were included in the “low-frequency cybersex group” (*n* = 41 0 < score < 1).

An ANOVA of the factors of frequency of cybersex, craving for pornography, ratings of difficulties intimacy, and gender was performed. The analysis showed a significant frequency of cybersex effect *F*(2,266) = 314.84; *p* < 0.001, *F*(2,266) = 76.28; *p* < 0.001 and difficulties in intimacy effect *F*(1,266) = 12.18; *p* < 0.001. *Post hoc* comparisons of questionnaire measures in all groups were performed. The analysis showed that participants who had a high score on cybersex frequency had higher scores of craving for pornography and higher rates of difficulties in forming intimate relationship than those with low frequency of using cybersex.

Table 3 shows mean questionnaire ratings and comparisons using *t*-tests of ratings of cybersex, pornography, and difficulty in intimacy according to levels of use of cyberspace (low-frequency users compared with medium frequency users and high frequency).

**Table 3:** **Questionnaire Ratings according to levels of use of cyberspace (non-users, light users, moderate users, and heavy users)**.

	Ratings on frequency on the cybersex questionnaire	Comparison with low frequency cybersex group
“Low-frequency cybersex group” (*n* = 54)	0.74 (2.4)	
“Medium frequency cybersex group” (n = 172)	16.44 (10.6)	*t*(1,52) = 8.74; *p* < 0.001
“High frequency cybersex group” (n = 41)	54.95 (16)	*t*(1,39) = 21.27; *p* < 0.001

	**Ratings on craving for pornography questionnaire**	**Comparison with non-users**

“Low-frequency cybersex group” (*n* = 54)	37.35 (17.6)	
“Medium frequency cybersex group” (*n* = 172)	48.45 (27.5)	*t*(1,52) = 1.56; *p* = 0.125
“High frequency cybersex group” (*n* = 41)	89.22 (26.8)	*t*(1,39) = 9.22; *p* < 0.001

	**Ratings on intimacy questionnaire**	**Comparison with non-users**

“Low-frequency cybersex group” (*n* = 54)	10.78 (7.6)	
“Medium frequency cybersex group” (*n* = 172)	14.54 (8.6)	*t*(1,52) = 2.36; *p* < 0.05
“High frequency cybersex group” (*n* = 41)	19.83 (10.98)	*t*(1,39) = 5.05; *p* < 0.001

**Figure 2:**
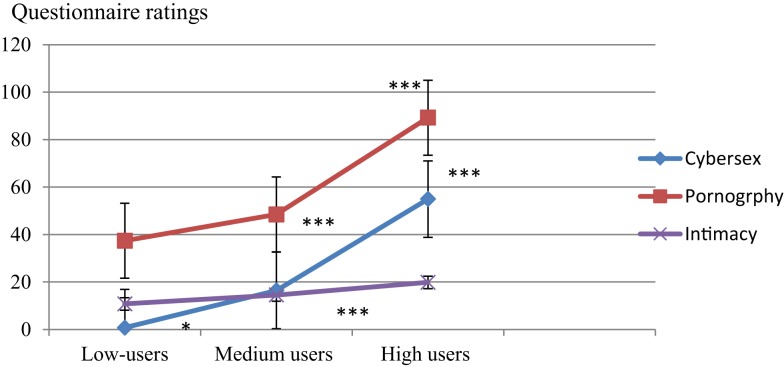
**Questionnaire ratings of frequency of use of cybersex, craving for pornography, and difficulties in intimacy in all participants**. *p < 0.05; **p < 0.01; ***p < 0.001.

Figure 2 demonstrates that higher levels of use of cyberspace were associated with higher levels of use of pornography and higher rates of difficulties in forming intimate relationships.

A Pearson correlational analysis between frequency of using cybersex, craving for pornography, and difficulties in forming intimate relationship scores was performed and it was found that frequency of using cybersex was positively correlated with craving for pornography (*r* = 0.68, *p* < 0.01). Second, frequency of using cybersex was positively correlated with difficulties in forming intimate relationship (*r* = 0.33, *p* < 0.01). Third, craving for pornography was positively correlated with difficulties in forming intimate relationship (*r* = 0.39, *p* < 0.01).

In men, ratings of difficulties in intimacy was positively correlated with cybersex ratings *r* = 0.47, *p* < 0.01 and with pornography ratings *r* = 0.48, *p* < 0.01 whereas in women, ratings of difficulties in intimacy was not correlated with cybersex ratings *r* = 0.11, *p* = N.S and with pornography ratings it only showed a trend of a positive correlation *r* = 0.22, *p* = 0.06.

Table 4 shows correlations on all questionnaires in all participants.

**Table 4:** **(A) Pearson’s correlations on all questionnaires in all participants; (B) Pearson’s correlations on all questionnaires in men; (C) Pearson’s correlations on all questionnaires in women**.

	Cybersex	Pornography
**(A)**
Cybersex		
Pornography	*r* = 0.68, *p* < 0.01	
Intimacy	*r* = 0.33, *p* < 0.01	*r* = 0.39, *p* < 0.01
**(B)**
Cybersex		
Pornography	*r* = 0.63, *p* < 0.0001	
Intimacy	*r* = 0.47, *p* < 0.01	*r* = 0.48, *p* < 0.01
**(C)**
Cybersex		
Pornography	*r* = 0.69, *p* < 0.0001	
Intimacy	*r* = 0.11, *p* = N.S	*r* = 0.22, *p* = 0.06

## Discussion

The results of this study showed that men had higher scores on measures of craving for pornography and frequency of using cybersex than women. These findings support previous evidence for sex differences in the use of pornography and online sexual behaviors between men and women see Ref. (30, 34) for review.

Previous research has found that both women and men use all types of online sexual activities but women were more interested in interactive online sexual activity while men were more interested in visual oriented online sexual activity (21, 35–38). In general, women found this use of sexual media acceptable or positive when associated with shared sexual activity. However, men reported more sexual enjoyment when pornography use was solitary; in those cases, women reported a partner’s solitary use was taking something away from the relationship (39, 40).

Gender has been found to be an important indicator of sexual attitudes and behaviors related to sexual explicit material found online (21, 41–44). Males were more likely than females to view erotic material online and offline and males go online at an earlier age to view sexual materials (45–48). Males most often report sexually explicit materials online to be arousing. While some females found these materials to be arousing, more reported the sexually explicit materials to be disturbing and disgusting (48). Women reported that the primary reason they used sexual media is as part of lovemaking with their partners or in response to requests by their partner. In general, women found this use of sexual media acceptable or positive when associated with a shared sexual activity. However, men reported more sexual enjoyment when pornography use was solitary; in those cases women reported a partner’s solitary use was taking something away from the relationship (39, 40). Females also reported feeling anger about online sexual materials (42), negatively compare themselves with online images (22), and often reported feelings of betrayal by their partners (49). The difference in reported frequency of using cybersex between men and women in our study may be since women feel fear of disclosure and feeling uncomfortable about admitting such activity. Second, since intimacy is an essential ingredient in cybersex which unlike pornography in general it is also characterized by chatting with a partner, participants may be jealously keeping discretion about this activity from their partner.

There could be several reasons why craving for pornography was higher in men than women in this study. Women prefer romantic fantasies and also look for intimacy and connection that is not provided by pornography whereas men look for short-term visual and graphic triggers for sexual arousal and prefer pornography. This pattern is supported by recent brain imaging studies that have demonstrated the differences between men and women in sexual arousal (50, 51). Hamann (51) examined brain activity with fMRI in men and women while they viewed sexually arousing photographs and neutral photographs. The primary finding was that the amygdala and hypothalamus exhibited substantially more activation in men than in women when viewing the same sexually arousing visual stimuli, presumably due to a stronger appetitive motivation or desire elicited by visual sexual stimuli. Furthermore, sexual activity in men is strongly related to psychological problems in daily life (28). Brand et al. (28) have found that in heterosexual males self-reported problems in daily life were linked to online sexual activities and these were predicted by subjective sexual arousal ratings of the pornographic material, global severity of psychological symptoms, and the number of sex applications used when being on Internet sex sites in daily life. Laier et al. (29) have also found that indicators of sexual arousal and craving to Internet pornographic cues predicted tendencies toward cybersex. Problematic cybersex users reported greater sexual arousal and craving reactions in response to pornographic cue presentation. However, the number and the quality with real-life sexual contacts were not associated to cybersex addiction. Finally, craving, sexual arousal rating of pictures, sensitivity to sexual excitation, problematic sexual behavior, and severity of psychological symptoms predicted tendencies toward cybersex addiction in Internet pornography users whereas being in a relationship, number of sexual contacts, satisfaction with sexual contacts, and use of interactive cybersex were not associated with cybersex addiction (30).

The finding of an association between craving for pornography and frequency of using cybersex is evident since those who started watching pornography have moved on to cybersex and vice versa and those websites advertise together both forms of sex media. The use of pornography is associated with difficulty in forming intimate relationship since pornography fills up a gap in the real world, and creates a virtual reality in which women always get satisfied and never complain. Cybersex enables those who have problems in attachment and avoid intimacy to form virtual relationships where warmth and affection and commitment are not required. An appealing feature of cybersex is that there is no requirement to perform the sexual act together so one does not fear performance anxiety. The use of sexual activity on the Internet affects sexual activity offline and there is evidence that some Internet users had abandoned or decreased their offline pornography consumption, while sexual compulsive users were found to increase their offline pornography consumption to a greater extent than did non-sexually compulsives (52).

Finally, sexual activity online negatively affected the relationship between men and women. Many studies showed that the consumption of Internet pornography threatens the economic, emotional, and relational stability of marriages and families (40, 53–61) see Ref. (25) for review. These studies indicated that pornography consumption, including cybersex, was significantly associated with decreased marital sexual satisfaction and sexual intimacy. Men and women perceived online sexual activity as threatening to a marriage as offline infidelity (56, 62).

The discovery that one of the partners is involved in sexual activity online leads to a re-evaluation of the relationship. A study conducted a web-based survey of 100 women whose partners used pornography showed that nearly one-third reported moderate to high levels of distress about their partner’s use of such material (53). They reported feeling as though their partners were not interested in making love to them, but during sexual intercourse were picturing the women they had seen in the pornography. They also felt their partners were less trustworthy, usually because he would keep the use a secret from them (even when they did not object to it). Nearly three-quarters reported feeling that the use negatively affected their self-esteem. Some felt they had failed their partners sexually; if they had been better sexual partners, their partners never would have had to turn to such material for sexual satisfaction. In this way sex on the Internet is quite often a mirror for dysfunctional sexual relationships at home and online as well (63). Schneider (24) has described how sexual addiction and compulsivity affected the patients, the spouse and the whole family. The survey respondents (93 women and 3 men) felt hurt, betrayal, rejection, abandonment, devastation, loneliness, shame, isolation, humiliation, jealousy, and anger, as well as loss of self-esteem. Being lied to repeatedly was a major cause of distress. Furthermore, cybersex addiction was a major contributing factor to separation and divorce of couples in this survey. Regarding the indirect impact on children of living in a home where a parent uses pornography, there is evidence that it increases the child’s risk of exposure to sexually explicit content and/or behavior (57). Children and youth who consume or encounter Internet pornography can have traumatic, distorting, abusive, and/or addictive effects. The consumption of Internet pornography and/or involvement in sexualized Internet chat can harm the social and sexual development of youth and undermine the likelihood of success in future intimate relationships (57). Schneider (24) has also reported adverse effects on the children including exposure to cyber porn and to objectification of women, involvement in parental conflicts, lack of attention because of one parent’s involvement with the computer and the other parent’s preoccupation with the cybersex addict, breakup of the marriage. In view of this abundant evidence for the damage of online pornography and cyberspace to couple and family life further research merits investigation on how to treat this modern outlet for sexual behavior.

## Limitations

Limitations, this study relied on ratings of subjective questionnaires which may result in variance of responses. Despite the promise of anonymity and confidentiality it is plausible that some of the responders have not fully disclosed the full information. Second, there may be other factors that are important in determining the effects of pornography and sex on intimacy and cybersex addiction that have not been investigated in this study. Thirdly, there was an unequal number of men and women with age difference between samples and this could limit the generalizability of the results. Finally, the Questionnaire on difficulties in intimacy by Marenco (33) has been widely used for research on psychosocial intimacy and for couple treatment but it needs further validation of reliability and validity in larger studies.

## Conclusion

In conclusion, the results of this study showed sex differences between men and women in their craving for pornography and frequency of using cybersex and that both craving for pornography and frequency of cybersex were associated with difficulty in forming intimate relationship. The reasons why people turn into cybersex are important, whether it is since passion has subsided over the years, or whether it is convenience, disappointment from past romantic relationships that lead into isolation and more. It is also important to know the reasons why people switched from pornography to cybersex and vice versa, whether it is the need for a partner or a need for stronger stimulation and arousal. A following study could also look at sexual preferences of men and women that may explain why for example some men or women use cybersex to fulfill homosexual activity. Finally, these studies have implications for treatment and sex therapy since a thorough understanding of the mechanisms and processes underlying compulsive sexual behavior are important for treating this disorder.

## Conflict of Interest Statement

The authors declare that the research was conducted in the absence of any commercial or financial relationships that could be construed as a potential conflict of interest.

## Appendix

### Intimacy questionnaire

Assign a number that best describes how you feel concerning each of the following statements. Base your answers on what has been true for you for the greater part of your life. What comes most quickly to your mind is usually the best answer.

0 = definitely not me, 1 = mildly disagree, 2 = neutral, 3 = mildly agree, 4 = definitely me.

**Table:**

1. You are concerned that if you truly reveal yourself to another person, s/he will leave
2. You fear that if someone really knew you that s/he would not like you
3. You have an uneasy feeling that people will smother you if you get too close
4. A parent physically or emotionally abandoned you in your childhood
5. You were teased or shamed for your feelings or needs when you were younger
6. You feel that one of your parents or significant caretakers was overly involved in your life
7. You would feel a sense of panic if you had a conflict with your partner and s/he pulled away
8. You would want to hide if your partner did a background check on you that was really on the mark
9. You find yourself needing more space in relationships once another person tells you that s/he really cares about you
10. You get angry when the person you’ve been involved with for six months says that s/he’s taking a vacation with friends that does not include you
11. You are comfortable showing your checkbook to your partner
12. You feel smothered when in the first few weeks of a relationship your partner wants you to call every day

### Scoring

1. Fear of abandonment (add your scores for questions 1, 4, 7, and 10) Total
2. Fear of exposure (add your scores for questions 2, 5, 8, and 11) Total
3. Fear of engulfment (add your scores for questions 3, 6, 9, and 12)

If you score higher than 10 on any of the three areas, this is a strong indication that this could be creating a block that prevents you from becoming more fully intimate with others.

## References

[1] Quadland MC. Compulsive sexual behavior: definition of a problem and an approach to treatment. J Sex Marital Ther (1985) 11(2):121–32. 10.1080/00926238508406078 4009729

[2] Garcia FD Thibaut F. Sexual addictions. Am J Drug Alcohol Abuse (2010) 36(5):254–60. 10.3109/00952990.2010.503823 20666699

[3] Karila L Wéry A Weinstein A Cottencin O Petit A Reynaud M Sexual addiction or hypersexual disorder: different words for the same problem? A review of the literature. Curr Pharm Des (2014) 20:1–10. 10.2174/13816128113199990619 24001295

[4] Rosenberg KP O’Connor S Carnes P Sex addiction: an overview. In: Rosenberg KP Feder LC, editors. Behavioral Addictions: Criteria, Evidence and Treatment. Burlington, MA: Elsevier Science (2014). p. 248–69.

[5] American Psychiatric Association. Diagnostic and Statistical Manual of Mental Disorders (DSM-5). 5th ed Washington, DC: American Psychiatric Publishing (2013).

[6] Carnes P Don’t Call It Love. New York, NY: Bantam Books (1991).

[7] Coleman-Kennedy C Pendley A Assessment and diagnosis of sexual addiction. J Am Psychiatr Nurses Assoc (2002) 8(5):143–51 10.1067/mpn.2002.128827

[8] Coleman E Raymond N McBean A Assessment and treatment of compulsive sexual behavior. Minn Med (2003) 86(7):42–7. 12921375

[9] Reid RC Assessing readiness to change among clients seeking help for hypersexual behavior. Sex Addict Compulsivity (2007) 14:167–86 10.1080/10720160701480204

[10] Carnes PJ. Sexual addiction and compulsion: recognition, treatment, and recovery. CNS Spectr (2000) 5(10):63–72. 17632453 10.1017/s1092852900007689

[11] Carnes P Out of the Shadows: Understanding Sexual Addiction. Center City Minnesota: Hazelden Information & Educational Services (2001).

[12] Black DW Kehrberg LL Flumerfelt DL Schlosser SS. Characteristics of 36 subjects reporting compulsive sexual behavior. Am J Psychiatry (1997) 154(2):243–9. 10.1176/ajp.154.2.243 9016275

[13] Carnes P Schneider JP. Recognition and management of addictive sexual disorders: guide for the primary care clinician. Lippincotts Prim Care Pract (2000) 4(3):302–18. 11271127

[14] Reid RC Carpenter BN Spackman M Willes DL. Alexithymia, emotional instability, and vulnerability to stress proneness in patients seeking help for hypersexual behavior. J Sex Marital Ther (2008) 34:133–49. 10.1080/00926230701636197 18224548

[15] Raymond NC Coleman E Miner MH. Psychiatric comorbidity and compulsive/impulsive traits in compulsive sexual behavior. Compr Psychiatry (2003) 44(5):370–80. 10.1016/S0010-440X(03)00110-X 14505297

[16] Reid RC Carpenter BN. Exploring relationships of psychopathology in hypersexual patients using the MMPI-2. J Sex Marital Ther (2009) 35(4):294–310. 10.1080/00926230902851298 19466668

[17] Miner MH Coleman E Center BA Ross M Rosser BR. The compulsive sexual behavior inventory: psychometric properties. Arch Sex Behav (2007) 36(4):579–87. 10.1007/s10508-006-9127-2 17192832

[18] Cooper A Griffin-Shelley E Introduction. The Internet: the next sexual revolution. In: Cooper A, editor. Sex and the Internet: A Guidebook for Clinicians. New York, NY: Brunner-Routledge (2003). p. 1–18.

[19] Krafka CD Linz D Donnerstein E Penrod S Women’s reactions to sexually aggressive mass media depictions. Violence Against Women (1997) 3:149–81 10.1177/1077801297003002004 12294812

[20] Paul P Pornified: How Pornography is Damaging Our Lives, Our Relationships, and Our Families. New York, NY: Times Books (2005).

[21] Cooper A Sexuality and the Internet: surfing into a new millennium. Cyberpsychol Behav (1998) 1(2):187–93 10.1089/cpb.1998.1.187

[22] Young KS. Internet sex addiction: risk factors, stages of development, and treatment. Am Behav Sci (2008) 52:21–37. 10.1177/000276420832133918045690

[23] Young K Are You Addicted to Cybersex? Bradford, PA: Center for Internet Addiction Recovery (2001).

[24] Schneider JP The impact of compulsive cybersex behaviours on the family. J Sex Marital Ther (2003) 18(3):329–54 10.1080/146819903100153946

[25] Manning JC The impact of Internet pornography on marriage and the family: a review of the research. Sex Addict Compulsivity (2006) 13(2–3):131–65 10.1080/10720160600870711

[26] Paul B Shim JW Gender, sexual affect, and motivations for Internet pornography use. Int J Sex Health (2008) 20:187–99 10.1080/19317610802240154

[27] Cooper A Delmonico DL Griffin-Shelley E Mathy RM Online sexual activity: an examination of potentially problematic behaviors. Sex Addict Compulsivity (2004) 11:129–43 10.1080/10720160490882642

[28] Brand M Laier C Pawlikowski M Schächtle U Schöler T Altstötter-Gleich C. Watching pornographic pictures on the Internet: role of sexual arousal ratings and psychological-psychiatric symptoms for using Internet sex sites excessively. Cyberpsychol Behav Soc Netw (2011) 14(6):371–7. 10.1089/cyber.2010.0222 21117979

[29] Laier C Pawlikowski M Pekal J Schulte FP Brand M. Cyber addiction: experienced sexual arousal when watching pornography and not real-life contacts makes the difference. J Behav Addict (2013) 2(2):100–7. 10.1556/JBA.2.2013.002 26165929

[30] Laier C Brand M Empirical evidence and theoretical considerations on factors contributing to cybersex addiction from a cognitive-behavioral view. Sex Addict Compulsivity (2014) 21:305–21 10.1080/10720162.2014.970722

[31] Brand M Young KS Laier C. Prefrontal control and Internet addiction: a theoretical model and review of neuropsychological and neuroimaging findings. Front Hum Neurosci (2014) 8:375. 10.3389/fnhum.2014.00375 24904393 PMC4034340

[32] Kraus SW Excessive Appetite for Pornography: Development and Evaluation of the Pornography Craving Questionnaire (PCQ-12). Ph.D. thesis, Advisor: Rosenberg H. Bowling Green State University (2013). Available from: http://www.researchgate.net/publication/256096657_Excessive_Appetite_for_Pornography_Development_and_Evaluation_of_the_Pornography_Craving_Questionnaire_(PCQ-12)

[33] Marenco A Intimacy Questionnaire: SOC SOC 103: College of the Canyons: Class Note (2014).

[34] Corley MD Hook JN Women, female sex and love addicts, and use of the Internet. Sex Addict Compulsivity (2012) 19:53–76 10.1080/10720162.2012.660430

[35] Cooper A Sexually compulsive behavior. Contemp Sex (1998) 32:1–3.

[36] Cooper A DL Delmonicob & R Burg Cybersex users, abusers, and compulsives: new findings and implications. Sex Addict Compulsivity (2000) 7(1–2):5–30 10.1080/10720160008400205

[37] Doring N The Internet’s impact on sexuality: a critical review of 15 years of research. Comput Hum Behav (2009) 25:1089–101 10.1016/j.chb.2009.04.003

[38] Flood M Young men using pornography. In: Boyle K, editor. Everydaypornography. New York, NY: Routledge (2010). p. 164–78.

[39] Bridges A Morokoff P Sexual media use and relational satisfaction in heterosexual couples. Pers Relat (2010) 18(2):1–24.

[40] Schneider JP Effects of cybersex addiction on the family: results of a survey. Sex Addict Compulsivity (2000) 7:31–58 10.1080/10720160008400206

[41] Delmonico D Miller J The Internet Sex Screening Test: a comparison of sexual compulsives versus non-sexual compulsives. J Sex Marital Ther (2003) 18(3):261–76 10.1080/1468199031000153900

[42] Goodson P McCormick D Evans A. Searching for sexually explicit materials on the Internet: an exploratory study of college students’ behavior and attitudes. Arch Sex Behav (2001) 30:101–18. 10.1023/A:1002724116437 11329723

[43] Buzzell T Demographic characteristics of persons using pornography in three technological contexts. Sex Cult (2005) 9:28–48 10.1007/BF02908761

[44] Janghorbani M Lam TR. Sexual media use by young adults in Hong Kong: prevalence and associated factors. Arch Sex Behav (2003) 32:545–53. 10.1023/A:1026089511526 14574098

[45] Boies SC University students’ uses of and reactions to online sexual information and entertainment: links to online and offline sexual behavior. Can J Hum Sex (2002) 11:77–89.

[46] Johansson T Hammar’en N Hegemonic masculinity and pornography: young people’s attitudes toward and relations to pornography. J Mens Stud (2007) 15:57–70 10.3149/jms.1501.57

[47] Peter J Valkenburg PM. Adolescents’ exposure to sexually explicit materials on the Internet. Commun Res (2006) 33:178–204. 10.1111/j.1365-2850.2011.01815.x 22073927

[48] Nosko A Wood E Desmarais S Unsolicited online sexual material: what affects our attitudes and likelihood to search more? Can J Hum Sex (2007) 16:1–10.

[49] Schneider JP Weissb R Samenowc C Is it really cheating? Understanding the emotional reactions and clinical treatment of spouses and partners affected by cybersex infidelity. Sex Addict Compulsivity (2012) 19:123–39 10.1080/10720162.2012.658344

[50] Hamann S Herman RA Nolan CL Wallen K. Men and women differ in amygdala response to visual sexual stimuli. Nat Neurosci (2004) 7(4):411–6. 10.1038/nn1208 15004563

[51] Hamann S. Sex differences in the responses of the human amygdala. Neuroscientist (2005) 11(4):288–93. 10.1177/1073858404271981 16061516

[52] Daneback K Ross MK Mansson S-A Characteristics and behaviors of sexual compulsives who use the Internet for sexual purposes. Sex Addict Compulsivity (2006) 13:53–67 10.1080/10720160500529276

[53] Bridges AJ Bergner RM Hesson-McInnis M. Romantic partner’s use of pornography: its significance for women. J Sex Marital Ther (2003) 29:1–14. 10.1080/713847097 12519658

[54] Cooper A Galbreath N Becker MA. Sex on the Internet: furthering our understanding of men with online sexual problems. Psychol Addict Behav (2004) 18(3):223–30. 10.1037/0893-164X.18.3.223 15482077

[55] Stack S Wasserman I Kern R Adult social bonds and use of Internet pornography. Soc Sci Q (2004) 85:75–88 10.1111/j.0038-4941.2004.08501006.x

[56] Whitty MT. Pushing the wrong buttons: men’s and women’s attitudes towards online and offline infidelity. Cyberpsychol Behav (2003) 6(6):569–79. 10.1089/109493103322725342 14756923

[57] Black C Dillon D Carnes S Disclosure to children: hearing the child’s experience. Sex Addict Compulsivity (2003) 10:67–78 10.1080/10720160309045

[58] Corley MD Schneider JP Sex addiction disclosure to children: the parents’ perspective. Sex Addict Compulsivity (2003) 10:291–324 10.1080/713775416

[59] Mitchell KJ Finkelhor D Wolak J The exposure of youth to unwanted sexual material on the Internet: a national survey of risk, impact and prevention. Youth Soc (2003) 34(3):330–58 10.1177/0044118X02250123

[60] von Feilitzen C Carlson U editors. Children in the new media landscape: games, pornography, perceptions. Children and Media Violence-Yearbook 2000. Goteborg: The UNESCO International Clearinghouse on Children and Violence on the Screen at Nordicom (2000).

[61] Greenfield PM Inadvertent exposure to pornography on the Internet: implications of peer-to-peer file-sharing networks for child development and families. App Dev Psychol (2004) 25:741–50 10.1016/j.appdev.2004.09.009

[62] Cooper A Morahan-Martin J Mathy RM Maheu M. Toward an increased understanding of user demographics in online sexual activities. J Sex Marital Ther (2002) 28:105–29. 10.1080/00926230252851861 11894795

[63] Bergner RA Bridges AJ. The significance of heavy pornography involvement for romantic partners: research and clinical implications. J Sex Marital Ther (2002) 28(3):193–206. 10.1080/009262302760328235 11995598

